# Prognostic value of the coronary artery calcium score in suspected coronary artery disease: a study of 644 symptomatic patients

**DOI:** 10.1007/s12471-019-01335-7

**Published:** 2019-10-25

**Authors:** D. Rijlaarsdam-Hermsen, M. S. Lo-Kioeng-Shioe, D. Kuijpers, R. T. van Domburg, J. W. Deckers, P. R. M. van Dijkman

**Affiliations:** 1 Haaglanden Medical Centre Bronovo, The Hague, The Netherlands; 2 Erasmus Medical Centre, Rotterdam, The Netherlands

**Keywords:** Coronary artery calcium score, Symptomatic, Stable chest pain, Prognosis, Coronary artery disease

## Abstract

**Aim:**

The long-term value of coronary artery calcium (CAC) scanning has not been studied extensively in symptomatic patients, but was evaluated by us in 644 consecutive patients referred for stable chest pain.

**Methods:**

We excluded patients with a history of cardiovascular disease and with a CAC score of zero. CAC scanning was done with a 16-row MDCT scanner. Endpoints were: (a) overall mortality, (b) mortality or non-fatal myocardial infarction and (c) the composite of mortality, myocardial infarction or coronary revascularisation. Revascularisations within 1 year following CAC scanning were not considered.

**Results:**

The mean age of the 320 women and 324 men was 63 years. Follow-up was over 8 years. There were 58 mortalities, while 22 patients suffered non-fatal myocardial infarction and 24 underwent coronary revascularisation, providing 104 combined endpoints. Cumulative 8‑year survival was 95% with CAC score <100, 90% in patients with CAC score >100 and <400, and 82% with CAC score ≥400 Agatston units. Risk of mortality with a CAC score >100 and ≥400 units was 2.6 [95% confidence interval (CI) 1.23–5.54], and 4.6 (95% CI 2.1–9.47) respectively. After correction for clinical risk factors, CAC score remained independently associated with increased risk of cardiac events.

**Conclusions:**

Risk increased with increasing CAC score. Patients with CAC >100 or ≥400 Agatston units were at increased risk of major adverse cardiac events and are eligible for preventive measures. CAC scanning provided incremental prognostic information to guide the choice of diagnostic and therapeutic options in many subjects evaluated for chest pain.

## What’s new?

The coronary artery calcium (CAC) score provides a quantitative assessment of the overall atherosclerotic burden, a measure not readily available with other forms of testing. We studied the long-term prognostic value of CAC scanning in a consecutive series of 644 men and women with stable chest pain but without previous cardiovascular disease.

A graded relation was found between CAC score and prognosis. The increase in risk was graded, and thus with larger scores, the risk became higher.

Both CAC score >100 as well as ≥400 Agatston units were associated with an increased risk of mortality [relative risk (RR) 2.6, 95% confidence interval (CI) 1.2–5.5 and RR 4.6, 95% CI 2.1–9.5, respectively] as well as with the occurrence of other major cardiac events. This association was not modified by correction for type of chest pain and risk factors.

Subjects with CAC score ≥400 units, constituting about 25% of the patients, were at highest risk with a rate of death or myocardial infarction in the order of 3% per year and would qualify for preventive medical treatment. Subjects with a CAC score between 100 and 400 units should be considered for primary preventive measures given their increased mortality.

Thus, application of the CAC score provided relevant prognostic information in addition to the usual clinical risk characteristics in patients suspected of coronary artery disease, and its application could guide the subsequent choice of diagnostic and therapeutic options in many such subjects.

## Introduction

Conventional coronary angiography is the reference standard for the diagnosis of coronary artery disease (CAD), but its diagnostic yield is low [1]. This is not only true with regard to the actual diagnostic findings: many subjects undergoing coronary angiography exhibit findings that are considered normal. But the low return is also reflected in the lack of therapeutic consequences associated with the procedure. Only about 50% of abnormal diagnostic coronary angiographies in patients with stable chest pain are followed by revascularisation [2]. Coronary angiography using computer tomography (CTCA) has been suggested as an alternative and less invasive diagnostic technique. Still, randomised studies comparing CTCA with functional testing in suspected CAD found no differences in outcomes between these two approaches [3, 4].

The detection of coronary artery calcium (CAC) by non-contrast CT scanning could represent an alternative diagnostic and prognostic tool. The CAC score provides a quantitative assessment of the overall atherosclerotic burden, a measure not readily available with other forms of testing [5]. The CAC score has been shown to reliably predict events among various categories of subjects studied. Among asymptomatic subjects, CAC scanning improved prognostic classification [6]. In symptomatic individuals, the absence of CAC has been shown to identify subjects at low risk for cardiovascular disease (CVD) and cardiovascular events, precluding the need for further downstream testing [7].

The long-term worth of CAC scoring in patients with symptoms suggestive of CAD has not been studied extensively [8]. We therefore investigated the prognostic value of CAC scanning in a consecutive series of 644 men and women with stable chest pain.

## Methods

### Patients

From December 2004 to May 2011, patients consecutively referred for the evaluation of their chest pain underwent a CAC scan. Symptoms were classified as typical, atypical or non-specific. Patients with a history of CVD were excluded. Because previous research has shown that both the short- and long-term prognosis of patients with a CAC score of zero is excellent, such patients were excluded from the current analysis [7, 9].

The study complied with the Declaration of Helsinki and was approved by the local research ethics committee.

### Risk factors

Hypertension was defined as systolic blood pressure >140 mm Hg and/or diastolic blood pressure >90 mm Hg, or treatment for hypertension. Dyslipidaemia was defined as total cholesterol >6.0 mmol/l or treatment for dyslipidaemia. Diabetes mellitus was diagnosed when haemoglobin A1c was ≥48 mmol/l, fasting plasma glucose >7.0 mmol/l, a random plasma glucose >11 mmol/l or when glucose-lowering therapy was used. Smoking was self-reported. Family history of premature CAD was defined as a history of myocardial infarction, percutaneous coronary intervention or revascularisation before age 60 years in a first-degree family member. Pre-test likelihood was determined on the basis of history, age and sex [10].

### Calcium score

A 16-row MDCT scanner (Sensation 16 Siemens, Erlangen, Germany) was used to acquire a volume set of data of the heart according to standard spiral protocol. CAC was measured by the Agatston method (Calcium scoring, Wizard workstation, Sensation 16, Siemens). The CAC score was classified as 0.1–100, 100–400 and ≥400 Agatston units.

### Follow-up

Survival was assessed in May 2016 by use of the national Civil Registry. Surviving patients received a questionnaire to collect information on cardiac events and procedures. Potential clinical events were adjudicated after review of pertinent hospital records. The date of the last known information from either source was used to calculate follow-up time. Five patients were lost to follow-up and excluded from analysis.

### Data analysis

Categorical variables are presented as absolute numbers and frequencies and continuous variables as mean ± SD. Endpoints included: (a) overall mortality, (b) mortality or non-fatal myocardial infarction and (c) the composite of mortality, myocardial infarction or coronary revascularisation by either coronary artery bypass grafting or percutaneous coronary intervention. In the latter analyses, 70 early (occurring within 1 year after inclusion) revascularisations were censored to minimise verification bias. Log-rank tests were used to compare different curves. The Kaplan-Meier method was used to assess event-free survival for outcomes of interest. Cox proportional hazard models were employed to adjust the association of the CAC score and adverse cardiac outcome for potential confounders. SPSS software (version 24.0, SPSS Inc., Chicago, IL, USA) was used for statistical analyses.

## Results

### Patients and follow-up

We included 644 patients, 320 women and 324 men. Their mean age was 63 (range 35–88) years. Details of the study population are presented in Tab. 1. Based upon their type of chest pain, 13% of the patients had a low, 72% an intermediate and 15% a high pre-test likelihood of CAD. In the low pre-test likelihood group 12% of the patients showed a CAC score ≥400, compared to 33% in the high pre-test likelihood group.

**Table 1:** Baseline patient characteristics and clinical assessment

Characteristic	Males	Females	Total
Gender (%)	324 (50)	320 (49)	644 (100)
Age, years	61 (11)	65 (10)	63 (11)
Body mass index, kg/m^2^	27 (4)	27 (4)	27 (4)
Current smoker (%)	67 (21)	40 (13)	107 (17)
Diabetes (%)	55 (17)	58 (19)	113 (18)
Family history of CAD (%)	137 (42)	150 (47)	287 (45)
Systolic BP, mm Hg	142 (19)	143 (20)	143 (20)
Hypertension (%)	142 (44)	164 (51)	306 (47)
Cholesterol, mmol/l	5.4 (1.2)	5.7 (1.3)	5.6 (1.2)
Dyslipidaemia (%)	84 (26)	94 (29)	178 (28)
Type of chest pain (%)			
non-anginal	160 (50)	157 (49)	317 (49)
atypical	88 (27)	120 (38)	208 (32)
typical	76 (23)	43 (13)	119 (18)
CAC score, Agatston units			
>0.1–100 (%)	116 (36)	146 (46)	262 (41)
100–400 (%)	109 (34)	107 (33)	216 (34)
≥400 (%)	99 (31)	67 (21)	166 (26)

Data are *n* (%) or mean ± SD

*CAD* coronary artery disease; *BP* blood pressure; *CAC* coronary artery calcium

During a median follow-up of 8 (range 5–12) years, 58 patients died, while 22 others suffered a non-fatal myocardial infarction. In addition, 24 patients underwent coronary revascularisation after at least 1 year following their first clinical examination. Thus, 104 patients experienced the combined endpoint.

### Coronary artery calcium scoring and prognosis

In 261 (41%) patients, the CAC score was between 0.1 and 100. A CAC score between 100 and 400 was found in 217 (34%) patients, and the CAC score was ≥400 Agatston units in 166 (26%) patients.

**Fig. 1:**
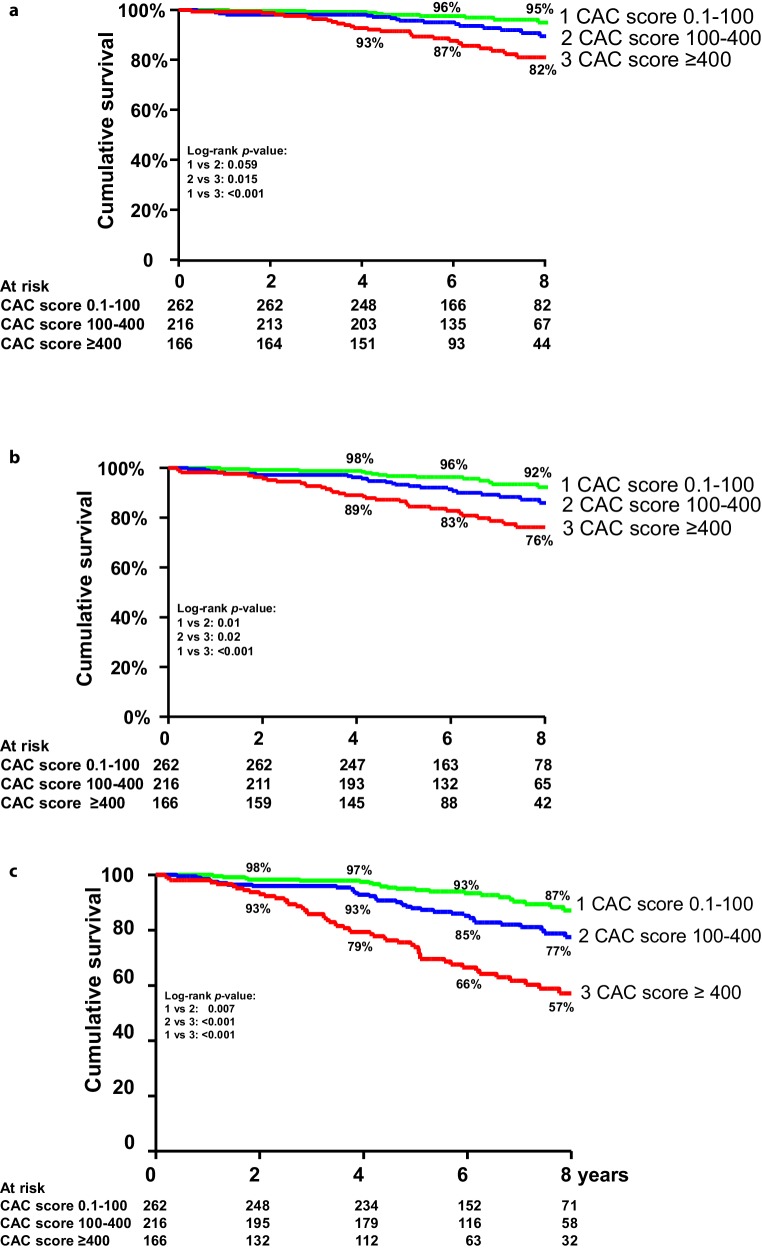
**a**–**c** Eight-year cumulative survival according to coronary artery calcium score categories. **a** Event-free survival mortality; **b** mortality and non-fatal myocardial infarction; **c** mortality, non-fatal infarction and (late) coronary revascularisation. *MACE* major adverse cardiac events

Cumulative 8‑year survival rate was 95% in patients with a low (<100) CAC score, 90% in patients with a CAC score between 100 and 400, and 82% in patients with a high (400 units or higher) CAC score. The survival of patients with a CAC score ≥400 was significantly (*p* < 0.01) lower than that of subjects with a lower score. The rate of the combined fatal and non-fatal events between the three groups of patients with varying degrees of coronary calcification was clinically relevant and statistically significantly different (Fig. 1).

In univariate analysis, a graded relation between CAC score and prognosis was found. Both CAC score >100 and ≥400 units were associated with increased risk of mortality [relative risk (RR) 2.6, 95% confidence interval (CI) 1.2–5.5 and RR 4.6, 95% CI 2.1–9.5, respectively] as well as with the occurrence of other cardiac events (Tab. 2). After correction for type of chest pain and risk factors, the associations between elevated CAC score and adverse events remained statistically significant for the combined endpoint. Other parameters associated with increased risk included higher age, male sex and positive family history of CAD.

**Table 2:** Unadjusted and adjusted association of coronary artery calcium score and adverse cardiac outcomes

Parameter	Univariate analysis		Multivariate analysis	
*Mortality* *n* *=58*				
Calcium score	RR	95% CI	RR	95% CI
0.1–100	Ref		Ref	
100–400	2.61	1.23–5.54	1.76	0.82–3.78
≥400	4.56	2.10–9.47	2.08	0.96–4.51
*Mortality and MI* *n* *=80*				
Calcium score	RR	95% CI	RR	95% CI
0.1–100	Ref		Ref	
100–400	2.41	1.29–4.50	1.79	0.95–3.37
≥400	4.15	2.26–7.60	2.31	1.22–4.41
*Mortality, MI, late revascularisation n* *=104*				
Calcium score	RR	95% CI	RR	95% CI
0.1–100	Ref		Ref	
100–400	2.06	1.22–3.48	1.76	1.03–2.99
≥400	4.49	2.71–7.45	2.80	1.80–5.37

*RR* relative risk; *C* confidence interval; *MI* myocardial infarction

## Discussion

Application of the CAC score provided relevant prognostic information in addition to the usual clinical risk characteristics in these men and women suspected of CAD. Increased risk was apparent from a CAC score of 100 Agatston units upwards. The increase in risk was graded; thus with larger scores the risk became higher. Subjects with a CAC score ≥400 units, constituting about 25% of the patients, were at highest risk with a rate of death or myocardial infarction in the order of 3% per year.

Although early establishment of a definitive anatomical diagnosis in subjects with chest pain seems desirable, two recent trials were unable to establish the benefit of such an approach. For instance, the Scottish Computed Tomography of the Heart (SCOT-HEART) trial, a study of low to intermediate risk patients, investigated the diagnostic value of CTCA [4]. The overall prevalence of obstructive CAD was 25%. Although CTCA changed the diagnosis of angina pectoris in 27% of the participants, altered recommendations for treatment in 18% as well as antianginal therapy in 9% (compared to 1% in the participants assigned to conventional clinical care), this was insufficient to lower the frequency and severity of symptoms or reduce the incidence of cardiac events. Diagnostic evaluations in the control group included stress electrocardiography (in 85%) and radionuclide stress imaging (in 10%). In the Prospective Multicenter Imaging Study for Evaluation of Chest Pain (PROMISE) trial, CTCA was not superior to functional testing, including nuclear stress imaging in 67%, stress echocardiography in 23% and exercise testing in 10% [3]. The results of these two large studies temper the over-enthusiastic use of CTCA as well as coronary angiography in the general screening of patients with chest pain. Such testing could perhaps be appropriate in specific subgroups of patients, but such categories have not yet been identified.

In patients with chest pain at intermediate risk, additional tests are often necessary to arrive at a robust diagnostic classification. Coronary angiography will be the method of ultimate choice in many. However, even the diagnostic use of coronary angiography has been found to be of limited clinical benefit [11]. Importantly, the difference between significant versus minor obstructive CAD is not straightforward and often fraught with uncertainty [12]. While coronary angiography may contribute in the identification of stable CAD, many stenoses thus identified may not be functionally significant. Evidence that either surgical or percutaneous coronary intervention improves the prognosis of unselected patients with stable chest pain without previous CVD is lacking [13].

Data about the effect of statin treatment on prognosis in subjects with high CAC scores are not conclusive. Arad et al. evaluated the treatment of 1005 asymptomatic subjects with CAC scores above the 80th percentile with atorvastatin 20 mg and antioxidant vitamins. In patients with CAC scores >400 a trend towards reduction of cardiovascular events was observed but did not achieve statistical significance [14]. A large observational study by Mitchell et al. showed that an increasing severity of CAC was associated with increased benefit from statin treatment for the prevention of major adverse cardiac events (MACE) with the greatest benefit in subjects with a CAC score >100. The estimated 10-year number needed to treat (NNT) for MACE for a CAC score >100 was 12 [15]. However, substantial differences between the statin and no statin group existed in CAC score and additional medication. Therefore, confounding could not be completely ruled out. In the Multi-Ethnic Study of Atherosclerosis (MESA) by Mortensen et al. mathematical models estimated the 10-year NNT for MACE for CAC score >100 as 28 assuming a 30% relative risk reduction with statin therapy [16]. Prospective randomised studies that can show the risk and benefit in CVD risk stratification based on CAC scoring, like the ongoing ROBINSCA trial, are very much needed [17].

The optimal strategy in suspected CAD therefore remains somewhat nebulous. Instead of establishing a diagnosis, prognostic evaluation of patients with suspected CAD surely considers merit. To this end, the exercise test has many proponents. The response to exercise provides a physiological measure of cardiac function at low cost. The Duke treadmill score, combining workload, angina and ischaemia into one numerical score, was found to add independent prognostic information to the clinical assessment in patients with suspected CAD [18]. However, the diagnostic employment of exercise testing in clinical practice is not uniform [3] and is given relatively low priority in the European guidelines [19].

The non-invasive detection of CAC is sensitive for the presence of significant CAD, and may be a useful adjunct in both diagnostic and prognostic work-up of patients with chest pain [20]. CAC is a continuous variable with increasing specificity with higher calcium scores indicating more severe atherosclerotic disease. However, while widely used for risk stratification and management guiding in asymptomatic individuals, the diagnostic and prognostic use of CAC scanning in patients with symptoms suggestive of CAD has been less extensively studied. Nevertheless, CAC scanning in conjunction with a clinically derived pre-test probability has been shown to reliably predict angiographic disease non-invasively [21].

In the evaluation of the additive prognostic performance of CAC score over CTCA, both CTCA percent stenosis and CAC were independently associated with increased incidences of MACE, while the addition of the CAC score to the model with clinical risk factors and CT stenosis improved predictive performance [22]. From a biological standpoint, the additive prognostic value of CAC over CCTA stenosis is not surprising. These techniques measure different aspects of CAD. CAC reflects calcified plaque burden, shown to be closely related to total coronary atherosclerotic development, whereas CTCA and coronary angiography focus on localised coronary luminal stenosis. A strong relationship has been established between CAC findings and coronary flow reserve. Increased frequency of coronary flow reduction was noted in patients with a CAC score ≥400 [23]. Such findings are supportive of a greater likelihood of important physiological changes with increasing anatomic atherosclerotic burden, assessed by the CAC score.

The incidence of true stable angina pectoris in symptomatic patients without a history of CVD is low [24]. Only a minority of men and women referred for evaluation of chest pain has angina pectoris resulting from obstructive CAD, and the low prevalence of significant CAD in patients with chest pain has been confirmed in many recent studies. For instance, significant CAD in the SCOT-HEART trial was present in only 25% [4]. In the PROMISE trial [3], only about 11% of all patients tested initially as ‘positive for CAD’. Among almost 400,000 patients undergoing elective coronary angiography following initial non-invasive examination, only 37% had obstructive CAD [1]. These findings illustrate the need for more efficient testing and better risk stratification of patients with chest pain. CAC scanning could well represent a useful initial test for this purpose, as additional tests are no longer needed in patients with either very low or very high CAC scores. In the recent prospective PROMISE trial [3], most patients experiencing clinical events presented with positive CAC scans, while less than half had functional stress test abnormalities. Although the abnormal functional test was more specific for CVD events, the discriminatory ability of CAC scanning proved to be a useful adjunct in the initial evaluation of patients with chest pain [25].

### Study limitations

By nature of the population studied, the number of fatal events in our study was quite small, and could have affected the power of the associations. In addition, the CAC score could have modified the diagnostic path, since an abnormal scan could have persuaded the physician to opt for coronary angiography and coronary revascularisation. However, this verification bias would have impacted mainly on the initial clinical course, and early coronary interventions were excluded from the current analysis. We did not investigate the effect of CAC scoring on downstream change of medication. This could have influenced outcome. In view of the favourable prognosis of patients with a CAC score of 0, these were excluded. Their inclusion would have increased the risk association of higher CAC scores with subsequent adverse cardiac events.

## Conclusion

In summary, in this study of 644 consecutive patients, CAC scanning provided significant incremental information on their 8‑year prognosis. Patients with a CAC score <100 units had a favourable prognosis. Both patients with a CAC score >100 or ≥400 Agatston units had increased risk of MACE and are eligible for preventive measures. Thus, CAC scanning provided incremental prognostic information and could guide the subsequent choice of diagnostic and therapeutic options in many subjects evaluated for chest pain.
